# A post-market, prospective, multi-center, single-arm clinical investigation of Phasix™ mesh for VHWG grade 3 midline incisional hernia repair: a research protocol

**DOI:** 10.1186/s12893-018-0439-7

**Published:** 2018-11-20

**Authors:** M. M. J. van Rooijen, A. P. Jairam, T. Tollens, L. N. Jørgensen, T. S. de Vries Reilingh, G. Piessen, F. Köckerling, M. Miserez, A. C. J. Windsor, F. Berrevoet, R. H. Fortelny, B. Dousset, G. Woeste, H. L. van Westreenen, F. Gossetti, J. F. Lange, G. W. M. Tetteroo, A. Koch, L. F. Kroese, J. Jeekel

**Affiliations:** 1 Erasmus University Medical Centre Rotterdam, Department of Surgery, Rotterdam, The Netherlands; 2 Imelda Hospital, Department of General Surgery, Bonheiden, Belgium; 3 University of Copenhagen, Bispebjerg Hospital, Department of Surgery, Copenhagen, Denmark; 4 Elkerliek Hospital, Department of Surgery, Helmond, The Netherlands; 5 Department of Surgery, University Hospital Lille, Lille, France; 6 Vivantes Klinikum Spandau, Department of Surgery, Berlin, Germany; 7 Department of Abdominal Surgery, University Hospital Leuven, Leuven, Belgium; 8 Department of Colorectal Surgery, University College London Hospital, London, UK; 9 Department of General and Hepatobiliary Surgery, University Hospital Ghent, Ghent, Belgium; 10 Wilhelminenhospital, Department of General, Visceral and Oncologic Surgery, Vienna, Austria; 11 Hôpital Cochin, Department of Digestive, Hepatobiliary and Endocrine Surgery, Paris, France; 12 Klinikum der Johann Wolfgang Goethe-Universität, Frankfurt am Main, Germany; 13 Isala Zwolle, Department of Surgery, Zwolle, The Netherlands; 14 Università di Roma Sapienza, Rome, Italy; 15 IJsselland Ziekenhuis, Department of Surgery, Capelle aan den Ijssel, The Netherlands; 16 Chirurgische Praxis Cottbus, Cottbus Area, Germany

**Keywords:** Incisional hernia, Complex hernia, Biosynthetic mesh, Mesh repair, Midline laparotomy, Surgical site occurrence, Complications

## Abstract

**Background:**

Incisional heia is a frequent complication of midline laparotomy. The use of mesh in hernia repair has been reported to lead to fewer recurrences compared to primary repair. However, in Ventral Hernia Working Group (VHWG) Grade 3 hernia patients, whose hernia is potentially contaminated, synthetic mesh is prone to infection. There is a strong preference for resorbable biological mesh in contaminated fields, since it is more able to resist infection, and because it is fully resorbed, the chance of a foreign body reaction is reduced. However, when not crosslinked, biological resorbable mesh products tend to degrade too quickly to facilitate native cellular ingrowth. Phasix™ Mesh is a biosynthetic mesh with both the biocompatibility and resorbability of a biological mesh and the mechanical strength of a synthetic mesh. This multi-center single-arm study aims to collect data on safety and performance of Phasix™ Mesh in Grade 3 hernia patients.

**Methods:**

A total of 85 VHWG Grade 3 hernia patients will be treated with Phasix™ Mesh in 15 sites across Europe. The primary outcome is Surgical Site Occurrence (SSO) including hematoma, seroma, infection, dehiscence and fistula formation (requiring intervention) through 3 months. Secondary outcomes include recurrence, infection and quality of life related outcomes after 24 months. Follow-up visits will be at drain removal (if drains were not placed, then on discharge or staple removal instead) and in the 1st, 3rd, 6th, 12th, 18th and 24th month after surgery.

**Conclusion:**

Based on evidence from this clinical study Depending on the results this clinical study will yield, Phasix™ Mesh may become a preferred treatment option in VHWG Grade 3 patients.

**Trial registration:**

The trial was registered on March 25, 2016 on clinicaltrials.gov: NCT02720042.

## Background

Incisional hernia (IH) is one of the most frequent complications after midline laparotomy, with incidences varying from 10 to 20%, and even higher percentages occur in high-risk groups [1, 2]. IH can lead to a high morbidity and reduces quality of life [3, 4]. Due to the high IH incidence rates, hernia repair surgery is one of the most frequently performed surgical procedures [5]. The aim of hernia surgery is to relieve symptoms, to prevent complications or to resolve acute complications.

There are several options for hernia repair, including primary simple suture repair, synthetic or biologic material placement, repair with relaxing incisions, component separation and use of musculofascial flaps, utilizing both open and laparoscopic approaches [6–8]. Synthetic mesh repair procedures, either open or laparoscopic, lead to fewer recurrences compared to primary repair; recurrences after mesh are 7.7% compared to 23.8% after primary closure [1, 3, 9, 10]. Improved outcomes are believed to be related to reduced tension on the fascial edges and sutures when mesh is used in hernia repair procedures. Despite reducing hernia recurrence rates, the use of synthetic mesh has been associated with complications in approximately 17% of patients. These complications include infection, pain, adhesions, fistulae and foreign body reactions including increased inflammation and/or connective tissue deposition [3, 11]. Especially complex and large abdominal wall defects continue to pose a challenge to surgeons, which are associated with recurrence rates of up to nearly 40% [12].

**Fig. 1:**
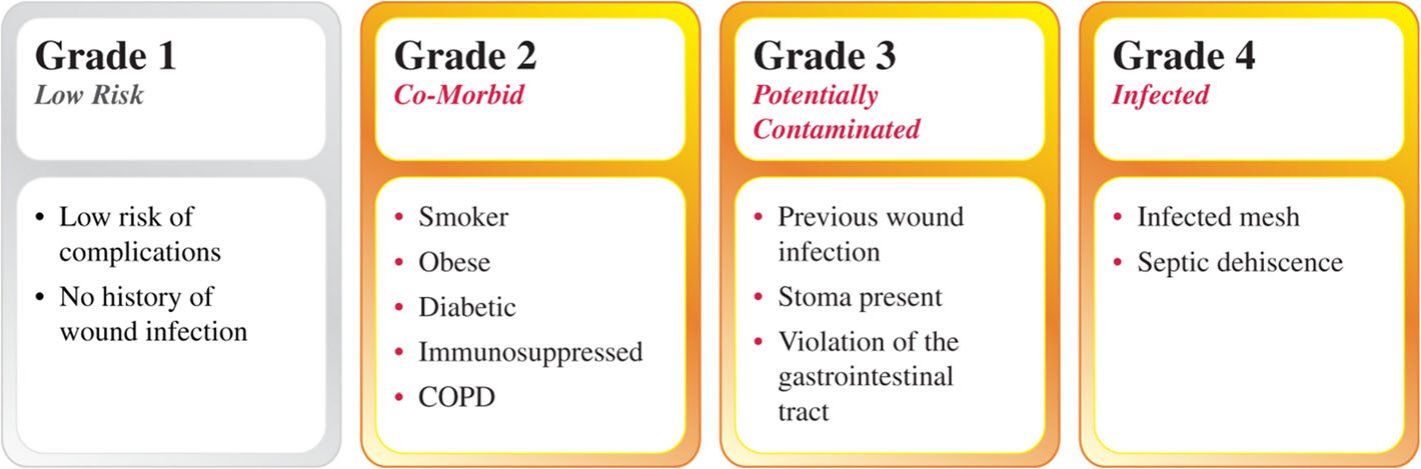
Hernia grading system: assessment of risk for surgical site occurrences [13]. (Reprinted from Surgery, 148(3), The Ventral Hernia Working Group, Incisional ventral hernias: Review of the literature and recommendations regarding the grading and technique of repair, 544-558, Copyright (2010), with permission from Elsevier)

It can be stated that synthetic mesh is more prone to infection than primary closure, and this poses a problem in potentially contaminated hernias like Ventral Hernia Working Group (VHWG) Grade 3 hernias [13] (Fig. 1). The success of the mesh repair is jeopardized by potential contamination due to complicating factors like previous wound infection, the presence of a stoma or violation of the gastro-intestinal tract.

The use of a biological tissue matrix has been advocated in (potentially) contaminated hernias, because of their ability to resist infection, milder inflammatory response and more orderly collagen deposition than non-resorbable, synthetic meshes [14–16]. Most often, biological meshes are derived from human, porcine or bovine dermis, and these materials have been processed to acellular sheets of collagen and elastin. The development of resorbable mesh products has faced challenges related to the rate of absorption with complications arising when the mesh product is resorbed too quickly. Rapid resorption does not support sufficient healing if structural reinforcement is diminished during the tissue repair period.

Therefore, some meshes contain chemicals to induce additional crosslinking in the graft. This slows down the degradation process, causing the mesh to retain its strength for a longer period of time [17]. However, crosslinking in the mesh reduces its biocompatibility; causing delayed cellular infiltration and neovascularization [17–19]. Ideally, a resorbable mesh should have a high ability to resist infections and retain its functional strength for a sufficient period of time to allow native cellular ingrowth tissue remodeling, maturation of collagen and gradual shift of mechanical load.

Phasix™ Mesh is a commercially available biosynthetic mesh. It is a slowly resorbable mesh prepared from poly-4-hydroxybutrate which has been studied for use as a biomaterial for different medical applications due its strength and flexibility, biocompatibility and desirable degradation times [20–22]. Phasix™ Mesh is comparable in performance to traditional polypropylene mesh when using standard measures of mechanical strength (suture pullout, tear and ball burst strength) [23, 24]. Preclinical implantation studies indicate that Phasix™ Mesh retains approximately 70% of its original strength at 12 weeks [23]. Absorption of the mesh material will be essentially complete in 12–18 months [24]. Given the long-term strength retention observed in preclinical studies, it is anticipated that Phasix™ Mesh may result in low recurrence and complication rates with minimal pain and discomfort when used for hernia repair.

### Rationale

From a general perspective, the current literature still is rather void of evidence-based guidelines regarding optimal choice of mesh. Simple, uncontaminated hernias are usually treated with synthetic mesh; biologic meshes are mostly used in potentially contaminated hernias, since post-operative mesh infection is anticipated.

Until now, the use of Phasix™ Mesh was studied primarily in patients up to VHWG Grade 2 [25].

Phasix™ Mesh has been studied prospectively until now in patients with a clean hernia site (CDC class I [26]) and up to VHWG Grade 2 [25, 27, 28]. A small retrospective study has also been conducted, showing positive results, but not elaborating on the exact contamination or size of the hernias [29]. Based on the data that will be gained from this clinical study, additional evidence may be provided with a view to optimal selection of hernia repair material in a population of higher risk. Since there is only limited knowledge on the treatment of VHWG Grade 3 patients, additional information is needed on safety and performance of the Phasix™ Mesh. Based on the combination of the features of the Phasix™ Mesh proven in previous clinical and non-clinical investigations, and based on evidence from the clinical study as described in this protocol, Phasix™ Mesh may become a preferred treatment option in VHWG Grade 3 patients.

## Methods

### Objectives

The objective of this study is to collect additional data on safety and performance of Phasix™ Mesh in subjects requiring VHWG Grade 3 midline incisional hernia repair. Among others, Surgical Site Occurrence (SSO), hernia recurrence, pain, infection, reoperation and adverse events will be collected for subjects with a VHWG Grade 3 hernia meeting the study inclusion and exclusion criteria.

### Design

The study has been designed as a post-market, prospective, single arm, multi-center, open-label study to collect data on performance and safety of Phasix™ Mesh in subjects with a VHWG Grade 3 midline hernia. This study will be conducted in 15 hospitals across Europe, which will each be allowed to include a maximum of 15 patients.

### Participants

Subjects with a VHWG Grade 3 incisional hernia scheduled for hernia repair are eligible for this study and will be asked for informed consent at the outpatient clinic. VHWG Grade 3 included, among others, previous wound infection after previous laparotomy (verified in the patient’s medical record), small bowel resection with anastomosis, take down of ileostomy with ileocolonic anastomosis, creation of a stoma, stoma present, jejunostomy, gastrectomy, and cholecystectomy. Patients with active infections, infected mesh, abscesses or active fistulas were not considered among patients with VHWG Grade 3. Patients solely at risk for an incidental enterotomy were not included in the population of which subjects could be drawn from.

#### Inclusion criteria

All subjects who meet the following criteria listed below can be enrolled in the study:

- Age 18 years or older
- Diagnosis of an incisional midline hernia
- VHWG Grade 3 hernia
- Size of hernia > 10 cm^2^, as measured intraoperatively
- Elective retro-rectus hernia repair
- Signed informed consent

#### Exclusion criteria

All subjects who meet the following criteria must be excluded from study enrolment:

Regarding the subject:

- Body Mass Index (BMI) > 35 kg/m^2^
- Peritonitis
- Use or suspected future use of chemotherapeutic medication during any part of the study
- Known human immunodeficiency virus (HIV) infection
- Cirrhosis of the liver and/or ascites
- Pregnancy, plans to become pregnant during the study period or current breastfeeding
- Alcohol/substance abuse problem or a relapse within 12 months of the screening visit
- Involvement in another interventional clinical study in the last 30 days prior to informed consent signature
- Life expectancy of less than 2 years at the time of enrollment
- Known sensitivity to Phasix™ Mesh or component materials (subjects with known allergies to tetracycline hydrochloride or kanamycin sulfate)
- Any condition that, in the opinion of the investigator, would preclude the use of the study device or preclude the subject from completing the follow-up requirements

Regarding ventral hernia:

- More than 4 previous repairs of the hernia under observation
- The hernia repair requires more than a single piece of mesh
- Intact permanent mesh adjacent to the current hernia to be repaired

Regarding surgery:

- American Society of Anesthesiology class 4 or 5
- Surgical technique requires surgical bridge repair
- Complete removal of existing mesh from a prior hernia repair (in the same affected area) is not possible
- The hernia repair requires intraabdominal mesh placement

### Study procedures

#### Screening

Subjects with a diagnosis of incisional midline hernia requiring surgical repair to close the defect who are presenting at the study site will be considered potential subjects for inclusion in this clinical study and should be pre-screened for study eligibility. If inclusion criteria are potentially met and no exclusion criteria are anticipated to be present at the time of pre-screening, the Investigator will invite the subject to participate in the study.

#### Informed consent

Subjects will be asked to sign a written informed consent form. A copy of the informed consent will be provided to the subject.

#### Eligibility

Final eligibility will be determined intraoperatively. Subjects who fail to meet eligibility criteria should be considered screen failures and will be treated per hospital standard of care. Reason for screen failure will be documented. Screen failures are not considered drop-outs, and hospitals will continue to include patients until the required sample size has received surgery.

### Intervention

All subjects will undergo an open ventral repair of the hernia. All intraoperative inclusion and exclusion criteria will be verified.

Subjects will be administered perioperative antibiotics according to hospital protocol. Subjects will be prepared to undergo hernia repair with Phasix™ Mesh. The general instructions for the use of Phasix™ Mesh are supplied by the manufacturer.

#### Surgical technique

The surgical technique will require retro-rectus placement (onlay is allowed as an exception when retro-rectus placement cannot be achieved), using slowly resorbable sutures, with or without Component Separation Technique (CST). The peritoneum should remain posterior to the mesh upon completion of mesh placement. The mesh may be cut to shape or size desired for each specific application. The mesh is to be positioned so its edges extend beyond the margins of the defect by at least 5 cm. It is recommended that the mesh is fixated at approximately 5–6 cm intervals (6–12 absorbable sutures) around the periphery of the mesh. Defect closure must be confirmed. All skin incisions will be closed with staples/sutures and wounds will be dressed with sterile occlusive dressings.

### Outcome parameters

#### Primary outcome

Primary outcome will be Surgical Site Occurrence (SSO) up to and including, the 3-month follow-up assessment. SSOs will be assessed by physical examination at each study visit through 3 months. SSO is defined as hematoma, seroma, surgical site infection, wound dehiscence, skin necrosis and fistula, all of which require intervention.

#### Secondary outcome

Secondary outcomes will be:

- Surgical Site Occurrence (SSO) after the 3-month follow-up assessment
- Surgical Site Infection (SSI) [26], is included in SSOs, but will also be analysed separately
- Hernia Recurrence rate (via physical exam, if uncertain via ultrasonography, CT or MRI)
- Pain at every follow-up point, measured with the Visual Analogue Scale (VAS)
- Device related adverse event incidence
- Rate of reoperation due to the index hernia repair
- Quality of Life assessments (Carolinas Comfort Scale™ [30]^1^ and EuroQol-5D (EQ-5D) [31])
- Surgical procedure time as measured from incision to closure (skin to skin)
- Return to work
- Length of hospital stay (day of index surgery until day of discharge, LOS)

To measure these outcomes, the following data will be gathered at different points in time, and saved in an electronic case report form:

#### Pre-operative data

- Demographic data (age, sex, race, ethnicity) and medical history
- Information regarding the inclusion and exclusion criteria
- Height and weight (calculated to a BMI)
- Length and width of hernia
- Wound assessment
  ○ signs of infection
  ○ status and location of potential previous mesh
  ○ signs of necrosis
- Pain medication usage
- Pain (measured with VAS), discomfort (measured with Carolinas Comfort Scale™) and quality of life (measured with EQ-5D)

#### Peri-operative data

- Information regarding the inclusion and exclusion criteria
- Intra-operative evaluation of wound and abdomen
- Intra-operative assessment and description of hernia
- Intra-operative assessment of complications, e.g. enterotomy
- Surgical procedure
- Mesh details
- Fixation details
- Wound closure

#### Post-operative data

The following data will be collected at fixed follow-up visits, namely at drain removal (if applicable, otherwise at discharge or at staple removal), 1 month, 3 months, 6 months, 12 months, 18 months and 24 months (Table 1):

- Wound assessment
  ○ signs of infection
  ○ status and location of potential previous mesh
  ○ signs of necrosis
- Hernia recurrence (diagnosed with physical exam, if uncertain via ultrasonography, or via CT/MRI)
- Adverse events
- Device failure/malfunction/defects
- Pain (measured with VAS)
- Discomfort (measured with Carolinas Comfort Scale™)
- Quality of life (measured with EQ-5D)

**Table 1:** Summary of procedures performed per visit

Study procedure	Screening and baseline	Index surgery	Drain removal/discharge	1, 3, 6 and 18 Month Visit	12 and 24 Month Visit	Early term
Describe study to potential subject	X					
Obtain informed consent	X					
Collect demographics and medical history	X					
Verify eligibility criteria	X	X				
Physical examination	X		X	X	X	X
Placement of device		X				
Pain Scale (VAS)	X		X	X	X	X
Carolinas Comfort Scale™	X			X	X	X
EQ-5D	X			X	X	X
Collect Adverse Events		X	X	X	X	X
Collect pain medications	X				X	

In addition, pain medication usage will be collected at 12 and 24 months follow-up.

#### Withdrawal/early termination

A subject is considered an Early Termination if discontinuation occurs after study treatment and before 24 months follow-up. The site will attempt to bring the subject back to the hospital to complete all Early Termination visit study procedures: Physical examination, Pain measured with VAS, Carolinas Comfort Scale™, EQ-5D and collect adverse events. Reason for subject discontinuation will be documented when possible.

### Sample size consideration

The expected rate of SSO at 3 months is 37% based on historical data (ranging from 21 to 53%) [32–35]. With 75 subjects, the accuracy of the estimated SSO will be ±11% (i.e. half of the width of the 95% confidence interval of the estimated rate of SSO is 11%). The study plans to enroll 85 subjects for follow-up. Anticipating on an attrition rate of about 10% after surgery, 75 subjects will be evaluable to assess the primary endpoint of Surgical Site Occurrence (SSO) at 3 months.

### Statistical analysis

There will be a modified intention-to-treat population (mITT), which consists of the subjects in whom Phasix™ Mesh has been implanted. The screen failures were not implanted, and therefore not used in the analysis. A per-protocol (PP) population may be created if there are subjects who have any major protocol deviations. However, all analyses will be primarily based on the mITT population.

Demographics and baseline characteristics will be summarized using the mITT population. Summary statistics for categorical variables will include frequency counts and percentages, and for continuous variables mean, standard deviation, minimum, median and maximum.

The primary endpoint is the SSO rate up to (including) 3 months (± 14 days) post device placement based on the mITT population. A 95% confidence interval will be reported for the SSO rate.

The SSO rate after 3 months, the hernia recurrence rates and surgical site infection rates until 1, 3, 6, 12, 18 and 24 months post device placement will be reported per visit along with their 95% confidence intervals based on the mITT population as secondary endpoints. Additionally, Kaplan-Meier analyses for the time from surgery to hernia recurrence and for the time from surgery to surgical site infection may be performed.

The secondary endpoints of VAS pain scale, Carolinas Comfort Scale™ and EQ-5D will be summarized based on the mITT population with mean, standard deviation, minimum, median and maximum presented by visit.

Device related adverse events will be tabulated by system organ class and preferred term. The number of subjects with a post procedure reoperation due to the index hernia repair will be presented by time intervals (until 1, 3, 6, 12, 18 and 24 months post device placement), surgical procedure duration of the index procedure (calculated as time of skin closure complete minus time of first incision) and length of hospital stay will be summarized descriptively. The time to return to work will be tabulated using summary statistics as well.

Safety parameters, such as adverse events, device deficiencies (mechanical failure, malfunction or defects), physical examination and pain medication, will be summarized using the mITT population.

Subgroup analyses will be performed by sex, sites (sites with few treated subjects can be combined) and other factors of interest.

No missing value imputation methods will be applied in any of the aforementioned analyses.

### Safety

In this study, Adverse Events (AE) are defined as any undesirable clinical event occurring in the abdominal wall or the abdominal space, as well as any other undesirable clinical events judged to be related to the study device or surgical procedure regardless of anatomical region, from time of implantation to end of study participation. Abnormal laboratory results are also to be considered as AEs if the results are accompanied by clinical signs or symptoms. The investigator will assess the relationship of an AE to the study device or procedure and categorize them as ‘definitely’, ‘possibly’ or ‘not related’.

An adverse device effect (ADE) is an AE related to the use of the mesh product implanted (e.g. insufficient or inadequate implantation, installation, operation or malfunction of the Phasix™ Mesh).

Serious adverse events (SAE) are the events that meet the definition of serious in the ISO 14155:2011.

All events will be followed to satisfactory resolution or stabilization.

The investigator is responsible for the detection and documentation of events meeting the criteria and definition of AE, ADE or SAE. All SAEs and investigator-judged device related AEs that occur, must be reported to the sponsor within 24 h of becoming aware of the event.

An independent safety monitoring committee will reassess safety of the study protocol and decide about potential adaptations if one of the following criteria are met:

- More than 4 device related SAEs within 3 months of Phasix™ Mesh implantation
- More than 1 device related recurrence within 3 months of Phasix™ Mesh implantation

The enrolment and treatment of new subjects are suspended until the impact of the study parameters (e.g. surgical technique, hernia size, mesh size, AE time-course) on the results is assessed. The follow-up for the subjects already treated continues.

Monitoring for accuracy and timely submission of data forms and compliance with the study protocol, meeting enrolment commitments and applicable regulations will take place by monitoring personnel.

### Ethics

This study will be conducted in accordance with the principles of the Declaration of Helsinki and Good Clinical Practice guidelines. The Medical Ethical Committee of the Erasmus Medical Center and the Institutional Review Board of every participating hospital have approved the protocol. Written informed consent will be obtained from all subjects. All study data will be recorded in electronic Case Report Forms provided to the investigational site. Site and subject numbers will be used to track subject information throughout the study.

The sponsor of the study has taken out an insurance policy for all participants of the study, in the case of any negative consequences experienced due to the study or the medical device.

The results of the study will be published, regardless of the outcome, either favourable or unfavourable, in a peer-reviewed journal and on clinicaltrials.gov, which is accessible for the public.

## Discussion

A major challenge in all hernia studies is the formulation of a clear definition on the severity or grade of the hernia. The difference between grade 3 and 4 hernias is not always clear, since the classification is more gradual than it seems. The definition for Grade 3 hernias used in this study is the same as the one of the Ventral Hernia Working Group in 2010, which excludes presence of infected mesh [13].

A discussion topic in this study is the absence of a control group. No standard treatment is registered for VHWG Grade 3 hernias. The standard treatment per hospital as a control group would not suffice, because 15 hospitals in Europe participate; this would have led to very heterogeneously treated control group with very heterogeneous results, insufficiently valid to compare to the performance of Phasix™ Mesh. Suture closure was considered as a control group, but this would have been disadvantageous for the patient because this has been proven to lead to more recurrences [1]. Also non-absorbable synthetic mesh was considered as a control group, because synthetic mesh placement reduces recurrences compared to suture closure or closure with the aid of biological mesh [36, 37]. However, synthetic mesh has been hypothesized to lead to a high infection rate due to the potential contamination present in VHWG Grade 3 hernia patients [38, 39]. Thirdly, the comparison with biological mesh is also hypothesized to be contra-indicated. Biological mesh has a high salvage rate when infected [40, 41], but has a higher recurrence rate than repair with synthetic mesh [37].

Because no standard treatment is recorded for VHWG Grade 3 hernias, comparing Phasix™ Mesh with synthetic mesh has been considered to be unethical, since the potential contamination of the hernia could cause complications when using a synthetic mesh. Comparing Phasix™ Mesh with just sutures (primary closure) would not be ethical either, due to the high recurrence rates associated with primary closure.

It was considered to compare Phasix™ Mesh with the treating surgeon’s standard of care for VHWG Grade 3 hernias in each participating hospital. However, due to the lack of consensus on what standard of care for VHWG Grade 3 hernias is, this would lead to a very heterogenous control group. This justifies the single-arm design of the study. Therefore, no randomization or blinding has been applied, leading to a possible higher risk of bias. If this study yields positive results, a large randomized controlled trial would be the next step in the exploration of Phasix™ Mesh augmentation in VHWG Grade 3 patients.

Due to the extensive inclusion- and exclusion criteria used in this study, and the specific goal of assessing safety and performance, the expected generalizability will be limited. VHWG Grade 3 patients are very specific patients with a high risk of developing an SSO. However, a study has shown that patients with only a history of infection after previous laparotomy without the other factors determining VHWG Grade 3, have a lower risk of developing an SSO. Therefore, it suggests a modified VHWG Grade 3 scale. It would be useful to analyse the results of the study described in this protocol between patients with only a previous infection after a previous laparotomy, and patients with one or more of the other factors determining VHWG Grade 3. Stratification for other confounders or effect modifiers, such as the presence of a stoma or the CDC wound classification could be of interest as well.

Surgeon skill is known to be an important predictor in surgical outcomes. Even though 15 different hospitals with 16 different surgeons participated, all surgeons have more than 10 years of experience in hernia surgery.

## Conclusion

This multicenter trial will collect additional data on safety and performance of Phasix™ Mesh in subjects with a VHWG Grade 3 midline hernia requiring surgical repair. Based on evidence from this clinical study Depending on the results this clinical study will yield, Phasix™ Mesh may become a preferred treatment option in VHWG Grade 3 patients.

## Abbreviations

ADE: Adverse Device Effect
AE: Adverse Event
BMI: Body Mass Index
CCS: Carolina Comfort Scale
CST: Component Separation Technique
CT: Computed Tomography Scan
EQ-5D: EuroQol-5D
HIV: Human Immunodeficiency Virus
IH: Incisional Hernia
LOS: Length of hospital Stay
mITT: Modified Intention-to-treat
MRI: Magnetic Resonance Imaging
PP: Per Protocol
SAE: Serious Adverse Event
SSI: Surgical Site Infection
SSO: Surgical Site Occurence
™: Trademark
VAS: Visual Analogue Scale
VHWG: Ventral Hernia Working Group

## Footnotes

1 The CAROLINAS COMFORT SCALE™ questionnaire was created by and is licensed from the Division of Gastrointestinal and Minimally Invasive Surgery of Carolinas Medical Center, North Carolina
